# WNK pathways in cancer signaling networks

**DOI:** 10.1186/s12964-018-0287-1

**Published:** 2018-11-03

**Authors:** Sachith Gallolu Kankanamalage, Aroon S. Karra, Melanie H. Cobb

**Affiliations:** Department of Pharmacology, The University of Texas Southwestern Medical Center, 6001 Forest Park Road, Dallas, TX 75390-9041 USA

**Keywords:** WNKs, OSR1, SPAK, STK39, Cellular signaling, Ion transport, Cancer, PI3K-AKT, TGF-β, And NF-κB

## Abstract

**Background:**

The with no lysine [K] (WNK) pathway consists of the structurally unique WNK kinases, their downstream target kinases, oxidative stress responsive (OSR)1 and SPS/Ste20-related proline-alanine-rich kinase (SPAK), and a multitude of OSR1/SPAK substrates including cation chloride cotransporters.

**Main body:**

While the best known functions of the WNK pathway is regulation of ion transport across cell membranes, WNK pathway components have been implicated in numerous human diseases. The goal of our review is to draw attention to how this pathway and its components exert influence on the progression of cancer, specifically by detailing WNK signaling intersections with major cell communication networks and processes.

**Conclusion:**

Here we describe how WNKs and associated proteins interact with and influence PI3K-AKT, TGF-β, and NF-κB signaling, as well as its unanticipated role in the regulation of angiogenesis.

## Plain English summary

WNKs are unique signaling kinases that bind and modify a host of cellular proteins to maintain homeostasis. As a consequence, these kinases are involved in the control of cancerous states. To better understand the growing picture of how WNKs affect human disease, we compiled this review to highlight signaling pathways often overlooked in WNK biology.

## Background

The with no lysine [K] (WNK) pathway is an ancient protein kinase signaling axis best known to regulate ion transport across cell membranes in mammals [1, 2]. However, it is an oversimplification to call this its primary role, or to imply that its role in ion transport regulation is completely understood. WNKs behave in many ways in many contexts, and to date there is no consensus on their general functions. WNKs are members of an unusual serine/threonine protein kinase family in which the position of the conserved catalytic domain lysine residue necessary for phosphoryl transfer is situated on the glycine-rich loop [3, 4]. This unique arrangement is accompanied by distinct structural and regulatory properties [5].

**Fig. 1:**
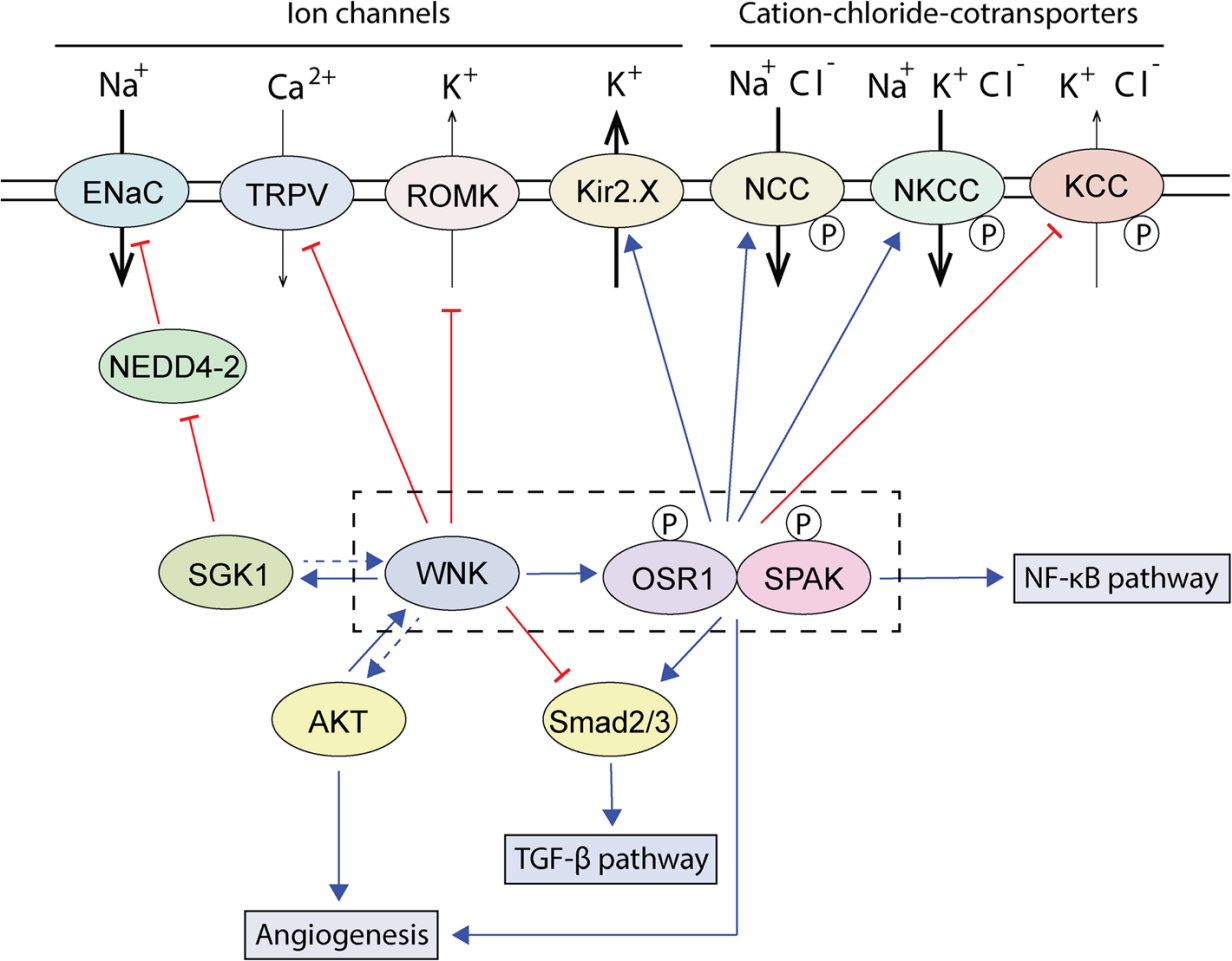
Overview of the WNK pathway and its connection to major cancer-associated signaling pathways discussed in the review. Activated WNK kinases phosphorylate and activate downstream substrate kinases OSR1 and SPAK, forming the core module of the WNK pathway (indicated by black dashed-line box). WNK signaling regulates several ion channels and cation-chloride-cotransporters via OSR1/SPAK-dependent and independent interactions. WNKs act both upstream and downstream of the PI3K-AKT pathway, which may affect AKT-mediated regulation of angiogenesis. WNKs exert direct inhibitory effects on the TGF-β signaling pathway, but OSR1 and SPAK can activate TGF-β signaling through Smad2/3. These opposing effects are likely at the root of context-dependent signaling between WNKS and TGF-β. SPAK is a known regulator of the NF-κB pathway, which may in turn be subject to upstream WNK signaling

An emerging area of study is the dependence of WNK pathway components in the progression of cancer, where the importance of these proteins is highlighted by their malleability. Activated WNK kinases phosphorylate and stimulate OSR1 and SPAK [6–8], serine/threonine kinases that phosphorylate and regulate the activity of a host of protein substrates [9–11] (Fig. 1). The extensive reach of the WNK signaling network encompasses major developmental processes and acute signal responses to changing cellular environments, underscoring the ramifications WNKs and their downstream factors have on disease conditions. Here, to better understand how WNK pathway functions affect cancerous states [12], we examine how WNKs exert regulatory influence on three major cancer-associated signaling networks: PI3K-AKT, TGF-β, and NF-κB signaling. Finally, we summarize some functions of WNK pathway components in angiogenesis, a process at the heart of cancer progression.

## Main text

### PI3K-AKT

The PI3K-AKT cascade is a well-studied oncogenic pathway subject to a diverse array of signaling inputs, most prominently insulin, and can influence numerous proteins, including WNK pathway components [13, 14]. In mice, acute insulin treatment results in increased phosphorylation of OSR1, SPAK, and the NaCl cotransporter (NCC) in the kidney; predictably, this effect is markedly diminished in WNK4 hypomorphic mice [15, 16]. PI3K-AKT signaling also activates the WNK-OSR1/SPAK-NCC cascade in a hyperinsulinemic db/db mouse model, providing key evidence of AKT-mediated kinase activation of WNKs [17]. AKT directly phosphorylates WNK1 at T60 (T58 in mouse and rat) in vitro, and in cells insulin-like growth factor (IGF)-1 can induce WNK1 T60 phosphorylation. Signaling from IGF-1 to WNK1 is blocked in HEK293 cells via treatment with PI3K inhibitors [18]. A similar relationship is also present in mouse embryonic stem (ES) cells; in ES cells lacking phosphoinositide-dependent kinase (PDK)-1, a protein required for AKT activation, IGF-1 is unable to induce WNK1 T60 phosphorylation [19, 20]. It is important to note, however, that this phosphorylation has not been shown to affect WNK1 kinase activity directly nor its cellular localization, and may instead influence protein-protein interactions [19]. Another study utilizing a phospho-specific antibody targeting AKT substrate phosphorylation sites following insulin treatment in 3T3-L1 cells identified WNK1 as a putative AKT substrate. Consistent with previous findings, insulin-stimulated phosphorylation of WNK1 is blocked by treatment with PI3K inhibitors and siRNA-mediated depletion of AKT. Although depletion of WNK1 does not affect insulin-induced glucose transport, it does increase insulin-induced proliferation of 3T3-L1 preadipocytes [18]. Both AKT and SGK1 phosphorylate WNK1 on this residue, and this phosphorylation is necessary to mediate insulin-induced endocytosis of ROMK [21] as well as for activation of SGK1 and subsequent stimulation of ENaC by WNK1 [22]. AKT-dependent WNK1 phosphorylation and enhanced WNK functions such as increases in phospholipase C-β signaling, angiogenesis, and cell migration have been reported by other studies as well [23–27]. Interestingly, WNK3 may also be a direct target of AKT [28]. WNK3 undergoes EGF-dependent phosphorylation in HEK293 cells and this phosphorylation is blocked by wortmannin, implying this is also a PI3K-AKT dependent event. PI3K-AKT signaling also affects the stability of WNK4. AKT phosphorylates Kelch-like 3 (KLHL3), which regulates WNK protein degradation along with Cullin3, a core component of an E3 ubiquitin ligase complex. AKT-mediated phosphorylation of KLHL3 reduces its interaction with WNK4, leading to sustained WNK4 expression [29]. Similar effects are seen with WNK1 and 4 mutations that also result in diminished interactions with KLHL3 and Cullin-3 [30–33].

In addition to acting as a target of the PI3K-AKT pathway, there is some evidence that WNK1 can act upstream of AKT. Hypotonic challenges can induce WNK1 T60 phosphorylation and cell proliferation in a rat smooth muscle cell line, and WNK1 overexpression also increases proliferation. WNK1 also increases the hypotonicity- induced activation of AKT and PI3K, whereas the depletion of WNK1 has opposite effects, suggesting that certain hypotonicity-induced cellular effects of WNK1 are mediated through the PI3K-AKT pathway [34] (Fig. 1).

Although currently there are no delineated mechanisms, there is ample evidence that WNK1 is important for cell migration in several cancer types, perhaps via the PI3K-AKT pathway. Acetyl-CoA medium-chain synthetase 3 (ACSM3) is a tumor suppressor in hepatocellular carcinoma (HCC). ACSM3 overexpression inhibits migration and invasion of the HCC cell lines HepG2 and SMMC7721, inhibits cancer metastasis in mouse xenografts, and its loss is associated with poor clinical outcomes [35]. ACSM3 overexpression also decreases the activation of AKT and phosphorylation of WNK1 at T60. Expression of an AKT construct blocked the tumor suppressor effects mediated by ACSM3, making it possible that the AKT-WNK1 axis promotes cell migration and invasion in HCC [27]. In non-small cell lung cancer (NSCLC) cells, the extracellular matrix component secreted protein acidic and rich in cysteine (SPARC) promotes cell migration and epithelial to mesenchymal transition (EMT): SPARC induces AKT activation and increases WNK1 phosphorylation at T60 [36]. Adding some credence to this is the observation that kynurenine (Kyn), which is produced by activated lung fibroblasts, facilitates migration and growth of lung cancer cells and also activates AKT and WNK1-T60 phosphorylation. The depletion of WNK1 in these cells inhibits migration in a manner similar to inhibition of AKT [26]. Because WNK1 T60 phosphorylation has been easier to detect with specific antibodies than activation loop phosphorylation, T60 phosphorylation has often been used as readout for WNK1 activity. It remains to be seen whether this specific post-translational modification directly influences WNK activity in cell migration. 

### TGF-β

WNKs also intersect with the transforming growth factor (TGF)-β pathway. TGF-β has both tumor suppressing and tumor promoting activities, although initial actions are often anti-proliferative [37]. Both WNK1 and WNK4 bind and phosphorylate Smad2/3, the downstream effectors of TGF-β signaling, but not Smad1/4. WNK1 depletion in HeLa cells decreases the total amount of Smad2, but increases phosphorylated Smad2 in cell nuclei, thus increasing localized TGF-β signaling [38]. Intriguingly, these finding are consistent with the recent discovery that WNK1 depletion increases the expression of TGF-β pathway genes in primary human endometrial cells [39].

SPAK mRNA and protein are upregulated in osteosarcoma patient samples, and knockdown of SPAK abolishes proliferation and downregulates invasion in osteosarcoma cell lines. SPAK depletion also decreases the phosphorylation of Smad2/3, suggesting that loss of SPAK antagonizes the tumor-promoting function of the TGF-β pathway in osteosarcoma [40]. There is also some evidence that OSR1 is a target of TGF-β signaling [41], and taken together these findings point to the high potential for WNK pathway components to influence tumor-related functions TGF-β signaling (Fig. 1).

### NF-κB signaling and SPAK

Nuclear factor Kappa B (NF-κB) is a well-known tumor promoter [42, 43]. Mouse *WNK1* has a putative NF-κB site on its promoter, suggesting that WNK1 expression in cells may increase following oncogenic activation [44]. However, within the WNK pathway, SPAK is the best characterized mediator of pro-inflammatory functions in an NF-κB-dependent manner [45] (Fig. 1). The Toll-like receptor 4, a transmembrane protein that stimulates NF-κB signaling, is known to lie upstream of SPAK-NKCC1 in rat choroid plexus epithelium [46], and may behave as a trigger for regulation of SPAK expression.

TNF-α induces demethylation of the *STK39* promoter and increases expression of SPAK in a manner that requires NF-κB binding to *STK39*; indeed, increased NF-κB enhances the expression of SPAK in cells [47]. SPAK expression is also upregulated by hyperosmotic stress, and this effect is dependent on NF-κB, which displays increased binding to the *STK39* promoter [48]. It is also notable that colon-specific (c)SPAK expression is upregulated in patients with ulcerative colitis, an inflammatory bowel disease that can be a contributing factor to colon cancer [49].

The potassium chloride cotransporter, KCC3, induces the expression of SPAK in cervical cancer cell lines and in HEK293 cells. KCC3 also upregulates NF-κB and MMP2 in HEK293 cells. It was suggested that NF-κB-induced SPAK activates the p38 pathway, leading to subsequent activation of MMP2 and promotion of cell invasion, although confirmation of this observation has not been reported. The depletion of SPAK also inhibits growth of xenograft tumors dependent on KCC3, supporting the relevance of SPAK in tumor growth within the context of NF-κB signaling [45].

SPAK functions not only downstream, but also upstream of NF-κB signaling, as mesangial cells primed with IgA immune complexes of SPAK knockout mice have decreased NF-κB and p38 signaling [50]. SPAK induces the production of pro-inflammatory cytokines, and SPAK knockout mice lack intestinal and renal inflammation and pro-inflammatory cytokine secretion compared to control mice [50–52]. However, SPAK is also activated by endotoxin and promotes nitric oxide production in mice [53]. SPAK increases the production of the anti-inflammatory cytokine interleukin 10 in T cells, leaving several open questions about the inflammatory consequences of SPAK activation [54, 55] (Fig. 1).

### Angiogenesis

Several components of the WNK pathway are implicated in angiogenesis, a process enhanced in many cancers to maintain blood flow to newly formed tumor tissue [12]. WNK1 knockout mice die before embryonic day 13 and display gross defects in cardiovascular development [56, 57]. Endothelial-specific WNK1 knockout mice also die mid-gestation with similar cardiovascular defects, consistent with the notion that WNK1-induced lethality is a result of failed development of the cardiovascular system and defective angiogenesis [57]. Whole body and endothelial-specific OSR1 knockout mice also fail to develop with similar cardiovascular and angiogenesis defects. Transgenic expression of an active mutant of human OSR1 in a mouse whole-body WNK1 knockout background rescues the developmental defects caused by the loss of WNK1, substantiating the importance of the WNK1-OSR1 axis in cardiovascular development [58].

Work done in our laboratory on cell lines and primary human endothelial cells has shown that WNK1 and OSR1 are required for angiogenesis in vitro. Depletion of WNK1 by siRNA inhibits cord formation in angiogenesis assays and also negatively affects cell proliferation and migration. Notably, WNK1 depletion leads to an anti-migratory gene expression pattern comprising inhibited expression of the mesenchymal transcription factors Slug, ZEB1, MMP2 and MMP9, and increased expression of thrombospondin-1. The depletion of OSR1 and of SPAK have different effects on endothelial cells: OSR1 is necessary for migration, whereas SPAK is necessary for proliferation. Interestingly, exogenous expression of Slug in WNK1-depleted cells partially rescues migration and endothelial cord formation, indicating WNK1 can act via OSR1, SPAK, and Slug to regulate angiogenesis [59] (Fig. 1).

In zebrafish, WNK1 depletion also blocks angiogenesis, indicating that its function in this process is conserved across species. Aberrations in zebrafish angiogenesis caused by the loss of vascular endothelial growth factor receptor (VEGFR)-2 can be partially rescued by ectopic expression of WNK1, although mutation of an AKT phosphorylation site on WNK1 (T60) blocks this partial rescue phenotype. Both VEGFR-2 and VEGFR-3 positively regulate the expression of *WNK1a*, one of the two major WNK1 isoforms in zebrafish, suggesting VEGFR pathway effects on angiogenesis promotion are enhanced in part through WNK1 [25] (Fig. 1).

## Conclusions and future directions

The WNK signaling axis is an ancient pathway that has been expanded, repurposed, and utilized in numerous contexts over evolutionary time. The need to learn more about its functions in cancer progression, in contrast, is a much more recent development. Currently, there are no clinically-approved drugs that target the WNK pathway being used to treat cancer. But given their unique structural organization, WNKs are attractive drug targets that may hold future promise [60, 61]; indeed, Yamada et al. have recently discovered an orally bioavailable pan-WNK inhibitor [61, 62]. A major challenge moving forward will be identifying reagents that are able to better discriminate specific phosphorylation events as well as WNK isoforms, due to the large number of signaling pathways involving WNKs. Compounds that inhibit OSR1 and SPAK activity have already shown promise as potential anti-cancer drugs [63–66]: Closantel, a SPAK inhibitor, inhibits cancer growth and angiogenesis when tested in a zebrafish model [65, 66], and Rafoxanide, another OSR1/SPAK inhibitor, inhibits CDK4/6 and is proposed as a potential therapeutic for skin cancer [63, 64]. Compounds that block binding between the RFXV/I motifs contained on WNKs and conserved C-terminal domains of OSR1/SPAK also hold promise as therapeutic interventions to inhibit the WNK pathway [60]. Notably, Mori et al. have discovered two such compounds, STOCK1S-50699 and STOCK2S-26016, that block these types of interactions [67]. This pharmacological evidence suggests the potential of targeting the WNK pathway in cancer.

While WNKs underlie several regulatory processes and phenomena, our goal in focusing on cancer signaling and angiogenesis was to provide a focused resource on an important emerging area of research. However, even through the lens of cancer progression, our current understanding of WNKs and their downstream targets make it clear that these factors display broad importance for cohesive cellular signaling and regulation.

## Abbreviations

ACSM3: Acetyl-CoA medium-chain synthetase 3
AKT: Protein Kinase B
EGF: Epidermal growth factor
EMT: Epithelial to mesenchymal transition
ENaC: Epithelial sodium channel
ES cells: Embryonic stem cells
HCC: Hepatocellular carcinoma
HEK293: Human embryonic kidney 293
IgA: Immunoglobulin A
IGF: Insulin-like growth factor
KCC3: Potassium-chloride cotransporter 3
KLHL: Kelch-like
Kyn: Kynurenine
MMP2: Matrix metalloproteinase 2
NCC: Sodium-chloride cotransporter
NF-κB: Nuclear factor Kappa B
NKCC1: Sodium-potassium-chloride cotransporter
NSCLC: Non-small cell lung cancer
OSR1: Oxidative stress responsive 1
PDK: Phosphoinositide-dependent kinase
PI3K: Phosphatidylinositol 3-kinase
ROMK: Renal outer medullary potassium channel
SGK: Serum/glucocorticoid regulated kinase
SPAK: SPS/Ste20-related proline-alanine-rich kinase
SPARC: Secreted protein acidic and rich in cysteine
STK39: Serine/Threonine kinase 39
TGF: Transforming growth factor
TNF-α: Tumor necrosis factor- α
VEGFR: Vascular endothelial growth factor receptor
WNK: with no lysine [K]
ZEB1: Zinc finger E-box-binding homeobox 1
